# Photonic zero mode in a non-Hermitian photonic lattice

**DOI:** 10.1038/s41467-018-03822-8

**Published:** 2018-04-03

**Authors:** Mingsen Pan, Han Zhao, Pei Miao, Stefano Longhi, Liang Feng

**Affiliations:** 1 Department of Materials Science and Engineering, University of Pennsylvania, Philadelphia, PA 19104 USA; 2 Department of Electrical Engineering, The State University of New York at Buffalo, Buffalo, NY 14260 USA; 3 Department of Electrical and Systems Engineering, University of Pennsylvania, Philadelphia, PA 19104 USA; 4 Dipartimento di Fisica, Politecnico di Milano and Istituto di Fotonica e Nanotecnologie del Consiglio Nazionale delle Ricerche, Piazza L. da Vinci 32, Milano, I-20133 Italy

## Abstract

Zero-energy particles (such as Majorana fermions) are newly predicted quasiparticles and are expected to play an important role in fault-tolerant quantum computation. In conventional Hermitian quantum systems, however, such zero states are vulnerable and even become vanishing if couplings with surroundings are of the same topological nature. Here we demonstrate a robust photonic zero mode sustained by a spatial non-Hermitian phase transition in a parity-time (PT) symmetric lattice, despite the same topological order across the entire system. The non-Hermitian-enhanced topological protection ensures the reemergence of the zero mode at the phase transition interface when the two semi-lattices under different PT phases are decoupled effectively in their real spectra. Residing at the midgap level of the PT symmetric spectrum, the zero mode is topologically protected against topological disorder. We experimentally validated the robustness of the zero-energy mode by ultrafast heterodyne measurements of light transport dynamics in a silicon waveguide lattice.

## Abstract (web summary)

Zero-energy states such as Majorana fermions could improve quantum computation, but they are not stable under strong coupling conditions. Here, Pan et al. demonstrate a photonic implementation of a topologically protected, non-Hermitian-enhanced, thus stable, zero mode in a non-Hermitian lattice.

## Introduction

Localized or propagative bound states with energy in the gap are commonplace in periodic systems with defects or disorder. However, bound states induced by defects are generally vulnerable to ambient randomness, impeding reliable manipulation of quantum coherence which is essential in many applications, such as in fault-tolerant quantum computation^1–5^. On the other hand, topologically protected edge states, such as those occurring in topological insulators and superconductors^6,7^, are intensively sought after for their protected surface conduction at the interface of two media of topologically distinct symmetries^8,9^. Acquiring states with robust energy against local or global perturbations are the main motivation for the study of such in-gap states. Among the investigated robust states, the dimensionless-bound state with its eigen energy pinned at the middle of a gapped band structure is referred to as the zero mode^10,11^. Examples of such zero-energy modes are the self-conjugated Majorana-bound states in *p*_*x*_ + *ip*_*y*_ superconductors^11^ that are regarded as the promising braiding anyons for the protective non-Abelian statistics, enabling fault-tolerant quantum error correction and computation^1–5^. In photonics and other wave systems, global symmetries and topological protection have inspired the design of engineered structures with unprecedented characteristics^9,12–28^, providing a platform to emulate topological states of matter such as topological insulators^13–16^, superconductor edge states, and zero-energy mode at a vortex^19^.

Robust localized states in the gap are known to arise when two crystals with different topological orders are interfaced, even in non-Hermitian systems^22–28^. It is commonly believed that these zero modes introduced by the topological defect site at the interface can only be protected by the invariants of two topologically distinct halves. These intriguing topologically protected characteristics become weak or even absent, if the topologically isolated defect mode couples with its surroundings, leading to deviations from the zero energy. One extreme configuration is to bring next to the defect site a crystal with exactly the same topological order, which eliminates the interface completely and thus the topological zero mode.

Here we address this challenge by demonstrating the existence of a genuine non-Hermitian topological defect state, despite the coupling of the defect mode to the surroundings with the same topological order. We investigate a paradigmatic model, a parity-time (PT) symmetric photonic lattice^29–33^, and demonstrate creation of the zero mode at the interface between two crystals with the same topological order but with distinct quantum phases, namely PT symmetry to broken PT symmetry . As a result of the non-Hermitian quantum phase transition, intriguing topological characteristics, such as the zero energy and topologically protected robustness against perturbations, are recovered.

## Results

### Theory

**Fig. 1:**
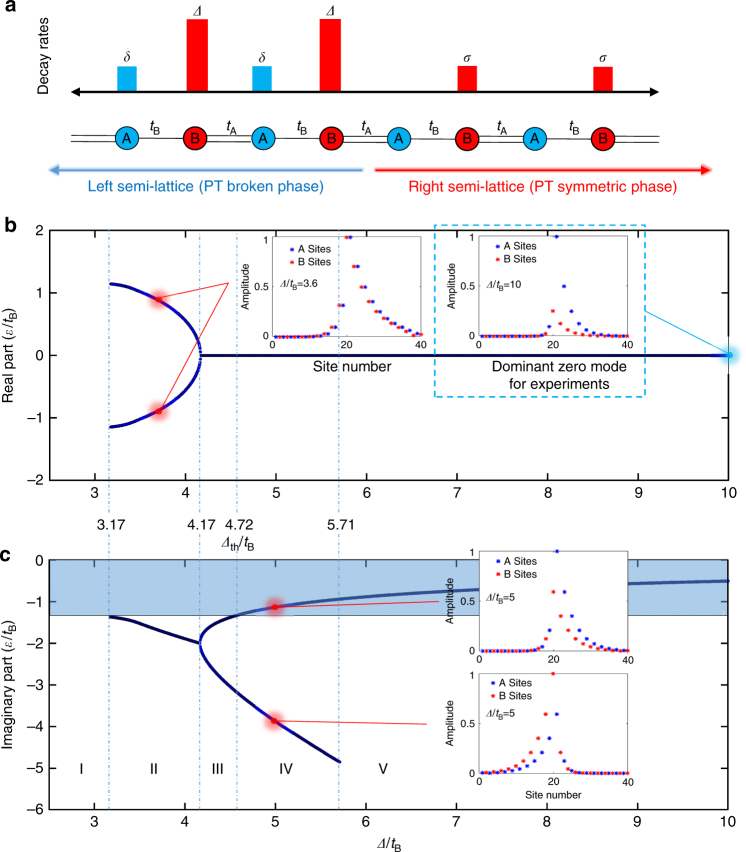
Bifurcation diagram of the interface modes in a Su–Schrieffer–Heeger (SSH) lattice. **a** Schematic of an interface realized by two dissipative SSH semi-lattices, where A sites have higher decay rates (*Δ* or *σ*) and B sites are associated with lower decay rates (*δ* or 0). Each semi-lattice is in the same topological order (i.e., we assume uniformly *t*_A_ > *t*_B_). **b**, **c** Bifurcation diagram of the interface states for *t*_A_/*t*_B_ = 2.5 and *σ* = 2.5*t*_B_, and for the right semi-array in the passive unbroken PT phase (Phase I: *δ* = 2.5*t*_B_). As *Δ*/*t*_B_ is increased, five distinct regions can be defined: Region I ($\Delta /t_{\mathrm{B}} \le$3.17), no interface states exist; Region II (3.17 ≤ *Δ*/*t*_B_ ≤ 4.17), there are two interface modes with opposite real part of energy, the same decay rate (imaginary part) and the same intensity distributions; Region III (4.17 ≤ *Δ*/*t*_B_ ≤ 4.72), there are two non-dominant zero-energy modes with different decay rates and intensity distribution; Region IV (4.72 ≤ *Δ*/*t*_B_ ≤ 5.71), one of the two zero-energy modes becomes dominant in the system; Region V (5.71 ≤ *Δ*/*t*_B_), there is only one dominant interface state, the other one becomes an unbound state whose field extends to the whole left lattice. The value of *Δ*/*t*_B_ that separates regions III and IV is the threshold value *Δ*_th_/*t*_B_ required to observe a dominant zero-energy mode in the dynamics. Note that for *Δ*$/t_{\mathrm{B}} \,> \,\delta /t_{\mathrm{B}} + 2(1 + t_{\mathrm{A}}/t_{\mathrm{B}})$=9.5, the left semi-array is in the fully broken passive PT phase (phase III), i.e., the interface of phase I and phase III semi-arrays can create a dominate zero-energy interface mode. The insets show a few distributions of mode amplitudes of interface states at a few values of *Δ*/*t*_B_. We did experiments on *Δ*/*t*_B_ = 10, circled by blue box in the picture. The colored area in **c** shows the region where an interface mode becomes dominant (i.e., lowest decay rate as compared to extended Bloch modes)

Our photonic lattice is based on a non-Hermitian extension of the Su–Schrieffer–Heeger (SSH) model^34^, where a tight-binding chain of coupled dimers is modulated with alternating onsite losses to realize a passive PT lattice^35,36^. Depending on the loss contrast in alternating sites, for the periodic lattice three different phases can be found^37^. We realize an interface between two semi-infinite lattices with the same topological order, which features a PT phase transition in a spatial domain (i.e., one semi-space is in the PT symmetric phase and the other is PT broken), as shown in Fig. 1a. The non-Hermitian phase transition at the interface decouples the real energy interaction of the two semi-lattices and introduces a robust zero-energy bound state, i.e., the reemergence of the topological defect state in spite of uniform topological order in the entire system. In other words, the non-Hermitian phase transition creates a zero-energy mode, which retains some topological protection but that would be prevented in the Hermitian limit. It is important to note that, while our system is loss dominant, it can perfectly reflect all the important PT symmetric features after averaging the loss modulation as background damping^35,36^.

After normalization with the background damping term, the Hamiltonian of the PT symmetric SSH model can be formalized in the momentum space as

1 $$H(k) = - i\gamma _0I + (t_{\mathrm{A}} + t_{\mathrm{B}}{\mathrm{cos}}(ka))\sigma _x + (t_{\mathrm{B}}{\mathrm{sin}}(ka))\sigma _y + i\gamma \sigma _z,$$

where *k* is the wavenumber in the momentum space, *a* is the lattice constant, *t*_A_ and *t*_B_ indicate the intra-dimer and inter-dimer couplings in the SSH model, −*iγ*_0_*I* is the background loss term in which *I* is a two-dimensional identical matrix, 2*γ* = *γ*_A_ − *γ*_B_ indicates the gain/loss contrast within one dimer (the decay rates of sites A and B in each dimer are represented by *γ*_A_ and *γ*_B_, respectively), and *σ*_*x,y,z*_ are the Pauli matrices. By filtering the background loss term in Hamiltonian (1), the energy dispersion can be calculated with these parameters, which reads

2 $$\varepsilon = \varepsilon _ \pm + i\gamma _0 = \pm \sqrt {t_{\mathrm{A}}^2 + t_{\mathrm{B}}^2 + 2t_{\mathrm{A}}t_{\mathrm{B}}{\mathrm{cos}}(ka) - \gamma ^2} ,$$

and sensitively depends on the value of *γ*. Depending on the value of loss contrast, the lattice can be found in three distinct phases (Supplementary Notes 1 and 2): unbroken PT phase for $\gamma < \left| {t_{\mathrm{A}} - t_{\mathrm{B}}} \right|$ (phase I), partially broken PT phase (phase II), and fully broken PT phase for $\gamma > \left| {t_{\mathrm{A}} + t_{\mathrm{B}}} \right|$ (phase III). Two semi-lattices in different phases realize a phase transition in a spatial domain^37^.

From a topological perspective, winding number and Chern number, etc. are often analyzed to determine the topological invariants in Hermitian systems. In a non-Hermitian system, such parameters cannot be well defined. However, the topological nature can be equivalently probed by the global Berry phase^24,37,38^, which, in our case, corresponds to the summation of complex Berry phase in both lower and upper bands. The Berry phase in each band can be calculated: $\varphi _{\mathrm{B}}^ \pm = {\oint}_k {A_ \pm } {\mathrm d}k$, where $A_ \pm = i\left\langle {u_ \pm } \right|{\mathrm d}/{\mathrm d}k\left| {\lambda _ \pm } \right\rangle$ is the Berry connection, and $\left\langle {u_ \pm } \right|$ and $\left| {\lambda _ \pm } \right\rangle$ are the normalized left and right eigenvectors of the Hamiltonian matrix *H*(*k*), respectively (see Supplementary Note 1 for detailed analysis), leading to

3 $$\varphi _{\mathrm{B}}^ \pm = \frac{{\varphi _0}}{2} \pm \frac{1}{2}{\oint}_{\phi _k} {{\mathrm{cos}}\gamma _k} \mathrm{d}\phi _k$$

where $\gamma _k = {\mathrm{arctan}}(\rho _k/i\gamma )$, $\rho _k = \left| {t_{\mathrm{A}} + t_{\mathrm{B}}{\mathrm{exp}}( - iak)} \right|$, and $\phi _k = {\mathrm{arg}}(t_{\mathrm{A}} + t_{\mathrm{B}}{\mathrm{exp}}( - iak))$. In Eq. (3), one can see both the intrinsic Berry phase in the Hermitian limit (i.e., $\varphi _0/2$) and the non-Hermitian-induced geometric phase.

Consequently, instead of being quantized, the Berry phase in each band becomes continuously varying due to the non-Hermitian-induced geometric phase. However, the global Berry phase remains quantized independent of onsite loss in our system (i.e., $\varphi _{\mathrm{B}}^ + + \varphi _{\mathrm{B}}^ - = \varphi _0$), revealing the same topological nature even in different quantum phases (Supplementary Fig. 1b). In other words, the non-Hermitian phase transition induced by increasing the gain/loss contrast is not expected to alter the topological phase of our system. Hence, while left and right halves of our photonic lattice possess different PT phases, they share the same topological order, the same as their Hermitian limits.

In another aspect, the interface sandwiched between the PT fully broken and unbroken phases has an effective decoupling in the real spectrum of the two sub-lattices. Due to the lack of paired state from the left lattice to compensate the energy splitting in the right lattice, the edge state in the right lattice reemerges as a localized zero mode at the parity-preserving termination site (i.e., the interface site)^37,39^. Because there is no real energy interaction at this interface, the zero-energy state can also be regarded as a state inherited from the flat band in the left lattice. As a result, a robust photonic zero mode emerges with the field strongly localized at the interface waveguide between two semi-lattices. In its Hermitian limit, the structure cannot support the zero mode because we interface two semi-lattices with the same topological order. Therefore, the zero-energy mode is driven by the PT phase transition at the interface rather than the different topological orders in the two semi-lattices. This non-Hermitian-enhanced protection of zero modes enriches the physical origin of the non-Hermitian zero modes in recent works^22–28^.

### PT phase transition in a passive system

Let us consider the interface shown in Fig. 1a, with modulated loss rates *δ*, *Δ* > *δ* in sub-lattices A and B of the left semi-array, and 0, *σ* in the right semi-array. Note that the background loss terms move the imaginary spectra below zero to different values in the two lattices, resulting in modified critical points for PT phase transitions. Bound states localized at the interface can be sought as discussed in Supplementary Note 2. Figure 1b shows a typical bifurcation diagram of energy curves (real and imaginary parts) of localized states for parameter values *t*_B_ = 0.0324 μm^−1^, *t*_A_ = 2.5*t*_B_, *δ* = 2.5*t*_B_, and *σ* = 2.5*t*_B_. *Δ* is chosen to be a controllable loss to tune the phase in the left lattice, and varies from *δ* to 2.5*t*_B_. The SSH chain consists of 20 dimers, with 10 dimers in each sub-lattice. The right semi-array is in the unbroken PT phase. When the left lattice is also in the unbroken PT phase (phase I–phase I interface), the whole system is in the same PT phase and topological order, and the states arising near the interface are the trivial Bloch states (region I in Fig.1b, c). Due to this strong semi-lattice coupling, no topological defect states can be found at the interface site. For a phase II to phase I interface, there are two non-zero energy interface modes with eigen-energies $\varepsilon _2 = - \varepsilon _1^ \ast$ (i.e., ${\mathrm{Re}}\left( {\varepsilon _1} \right) = - {\mathrm{Re}}\left( {\varepsilon _2} \right) \ne 0$), and with the same intensity distributions and decay rate (region II in Fig. 1b, c). For a phase III to phase I interface, the two sub-lattices are fully decoupled in their real energy spectra and two zero-energy states emerge. In this limit, the dominant zero-energy interface state that survives resembles the topologically protected edge state of the right lattice, and reduces to it as the loss term *Δ* is further increased thus fully decoupling the two semi-lattices (see the insets in Fig. 1b, c). The reemergence of topological edge states indicates that non-Hermitian phase transition enhances topological protection. With an increasing value of *Δ*, the decoupling begins to split the imaginary energies of the two interface states: one interface state can arise as a dominant state, the other one becomes more dissipative and finally sinks into the Bloch states in the left lattice. According to these behaviors of the spectrum, we divide the three PT phases into five regions: no existence of interface states (Region I), two non-topological interface states (Region II), two zero-energy non-dominant interface states (Region III), two zero-energy interface states, one of which becomes dominant (Region IV), and one single dominant zero-energy interface state (Region V). The four insets in Fig. 1b, c show the field amplitude distributions of the interface-bound states at different regions. With increasing decoupling of the two semi-lattices, the field becomes more localized at the interface. The dominant zero-energy mode acquires an enhanced topological protection ensured by non-Hermitian phase transition. As a matter of fact, we will demonstrate that the recovered zero-energy mode is insensitive to the topological disorder at the interface.

### Photonic zero mode at the PT phase transition interface

**Fig. 2:**
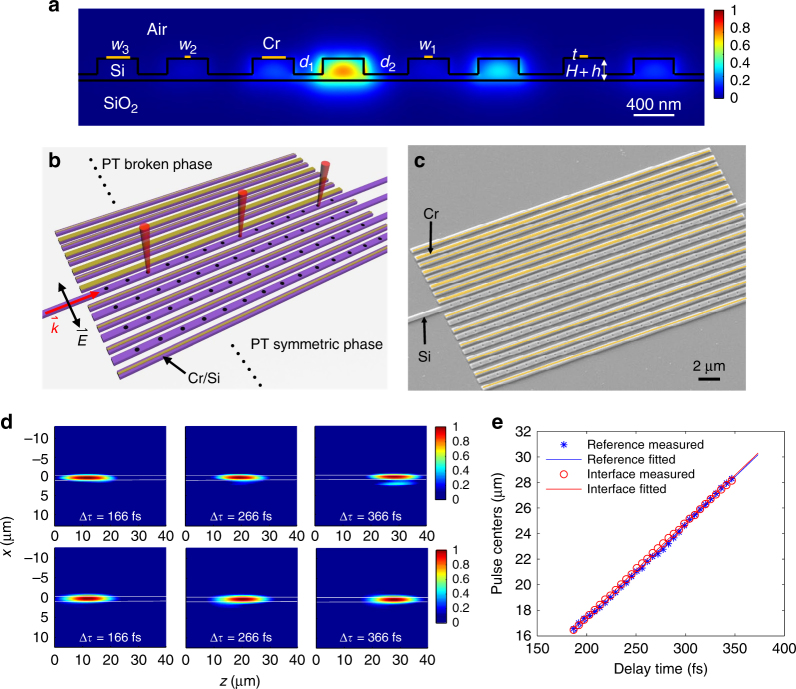
Observation of the zero mode on a photonic platform. **a** Cross section of the amplitude distribution in the tight-binding binary waveguide array to realize the phase transition and its induced zero mode at the interface. The width and height of the ridge waveguides are *W* = 400 nm, *H* = 150 nm and *h* = 70 nm, respectively. The coupling between waveguides is controlled by the separation between waveguide, where *d*_1_ = 300 nm for *t*_A_ = 0.0811 μm^−1^ and *d*_2_ = 450 nm for *t*_B_ = 0.0324 μm^−1^, for the operation wavelength of 1550 nm. The onsite damping is implemented by Cr depositions on top of waveguides with a uniform thickness of *t* = 20 nm but varied widths: *w*_1_ = 90 nm to create 2*γ* = 0.0811 μm^−1^ in the right PT symmetric semi-lattice, while *w*_2_ = 80 nm and *w*_3_ = 350 nm to create 2*γ* = 0.2838 μm^−1^ in the left PT broken semi-lattice. The waveguide lattice can then be viewed as a collection of coupled dimers, where *t*_A_ and *t*_B_ alternates. The eigen-mode simulation shows the field distribution of the interface state with an effective index of *n*_0_ = 2.37 at 1550 nm. **b** Schematic of the waveguide array sustaining the zero mode at the transition interface between the PT broken and symmetric semi-lattices, where the zero mode is excited by directly coupling quasi-TE polarized light into the interface waveguide. A set of periodic radiation holes are introduced on the top of the lossless waveguides to scatter light for far-field observations. **c** The SEM picture of the loss modulated PT waveguide array on an SOI platform, where pseudo yellow color denotes the Cr depositions on top of waveguides. **d** The same propagation lengths can be observed for the intensity distributions of the carried zero mode (upper panel) and a single waveguide control (bottom panel) at different time delays of 166, 266, and 366 fs. The middle/single waveguides are marked with solid lines. **e** The relation of the center of the wave packet traveling in time, where dots and lines represent raw measurement data and their corresponding linear fittings. The consistence between the interface mode (red) and the single waveguide mode (blue) evidently demonstrates the zero-energy characteristics

The photonic implementation of the interface structure is conducted using an array of coupled waveguides on a silicon on insulator (SOI) platform where each waveguide represents a site (A or B) in the SSH model as illustrated in Fig. 1a and Fig. 2a. In this passive system, the PT phase transition in the lattice is realized by different loss rates introduced through selective absorption of Cr depositions on top of the waveguides. The background of the schematic shows the field simulation on the cross section of the structure at an optical wavelength of 1550 nm. For easy excitation and test of robustness of the zero-energy interface state, we chose the strong loss regime *Δ*/*t*_B_ = 10, where the interface state dominates over scattered Bloch states. The photonic lattice is designed and implemented on an SOI platform (see Methods), as shown in Fig. 2b, c. To precisely characterize the zero-energy mode, it is necessary to assess a series of fundamental mode parameters including its group index and phase index, compared with the mode parameters of an unperturbed single waveguide. Here, we applied the heterodyne imaging technique^40^ (see Methods and Supplementary Note 3) to investigate the ultrafast transport dynamics to retrieve the quantitative characteristics of the interface state. The launched wave packet mainly couples into the interface waveguide. Since the Bloch states of the lattice are well suppressed by the introduced onsite losses, the propagation of the wave packet is robust against neighboring couplings and always remains confined in the interface waveguide (Fig. 2d upper panel and Supplementary Movie 1). As the wave packet propagates, the implemented single-waveguide excitation gradually evolves to the actual mode profile of the interface state evanescently extending to the adjacent dimer in the semi-lattice under the PT symmetric phase, consistent with the eigen-mode simulation in Fig. 2a.

To reveal the zero-energy characteristics of the interface state, a control study on a single unperturbed waveguide was also conducted with the same wave packet excitation (see Fig. 2d, bottom panel and Supplementary Movie 2). The results clearly demonstrate almost identical features between the interface state and the control waveguide at different time delays, regarding the pulse dispersion, shape, and duration. These ultrafast temporal measurements provide access to quantitative characteristics of the photonic modes. By sweeping the time-delay line with a constant time interval, the trace of the center of the wave packet traveling in time can be constructed for both the interface state and the single waveguide mode (Fig. 2e). The measured group index for the interface state is approximately *n*_g_ = 3.97, corresponding to an effective phase index of *n*_eff_ = 2.37, which are almost identical to the group and phase indices of the single waveguide of approximately *n*_g_ = 3.97 and *n*_eff_ = 2.37, respectively, evidently showing the zero-energy properties associated with the interface state. The slight discrepancy may arise from the waveguide index variation due to the Cr deposition across the photonic lattice, which barely brings variations of onsite energy and shifts the zero-energy point. It is important to note that a clear distinct feature of our waveguide lattice from previously observed topological interface state^22–28^ is that the topological order is uniform across the interface. This means that, in the Hermitian limit, i.e., assuming zero losses in all waveguides, the interface state disappears and the light pulse undergoes spatial spreading (discrete diffraction), which is confirmed in a control experiment using a lossless waveguide lattice (Supplementary Note 4).

### Robustness of the non-Hermitian-enhanced zero mode

**Fig. 3:**
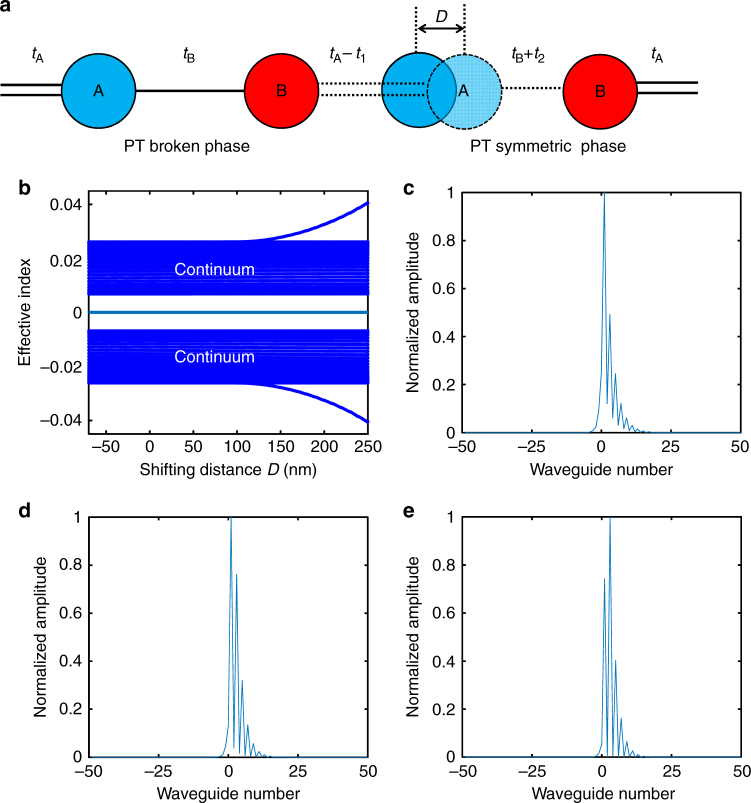
Robustness of the zero mode against a local perturbation. **a** Topological disorder by shifting the interface waveguide by a distance *D* toward the PT symmetric phase semi-lattice, which results in perturbation of local coupling parameters with –*t*_1_ and +*t*_2_. **b** Evolution of eigen-spectrum with respect to the shift of the interface waveguide. The interface state robustly resides at zero energy between the two continuum bands, while two defect states form due to strong coupling to the adjacent dimer when the shift of the waveguide is approximately more than 150 nm. Here the two edge states on the boundary of the lattice and the flat band introduced by PT symmetry-broken lattice have been removed to clearly show the interface state. **c**–**e** Mode amplitude distribution of the interface state with different topological perturbations corresponding to different shifts of the interface waveguide: **c** *D* = 0 nm; **d** *D* = 100 nm; **e** *D* *=* 200 nm

An important feature of the zero mode is its robustness against topological disorder. We consider a strong local perturbation of waveguide separation at the interface (Fig. 3a), which can even completely reverse the topological relation between intra- and inter-dimer couplings, i.e., *t*_A_ > *t*_B_ becomes *t*_A_ < *t*_B_ (see Methods). Remarkably, the zero mode is not destroyed and maintains its zero energy and mode profile, showing large fault-tolerance. It is clearly demonstrated in the spectrum (Fig. 3b) that the energy of the interface state maintains at zero, regardless of the strength of the introduced local topological disorder at the interface waveguide. As a result, the field remains localized in the interface waveguide region with exponential decay tails in both semi-lattices (Fig. 3c, d). Field localization at the interface is weakened, with a slower exponential decay into the PT symmetric semi-lattice, if the interface waveguide shifts more right, as its coupling to the adjacent dimer (i.e., $t_{\mathrm{B \prime}}$) grows. Nevertheless, if the value of $t_{\mathrm{B \prime}}$ is too high, i.e., the interface waveguide is too close to the adjacent dimer in the PT symmetric semi-lattice, they become strongly coupled to form a defect trimer. With this regard, the intensity peak slightly moves to the second B waveguide in the PT symmetric semi-lattice (Fig. 3d, e), but its associated zero energy is still protected through the phase transition. Additionally, two defect states emerge outside the continuum spectrum when the shift of the interface waveguide is larger than 150 nm (Fig. 3b). The energy of the two defect states is associated with relatively large damping coefficients, thereby attenuating much faster than the zero mode.

### Experimental validation of the topological robustness

**Fig. 4:**
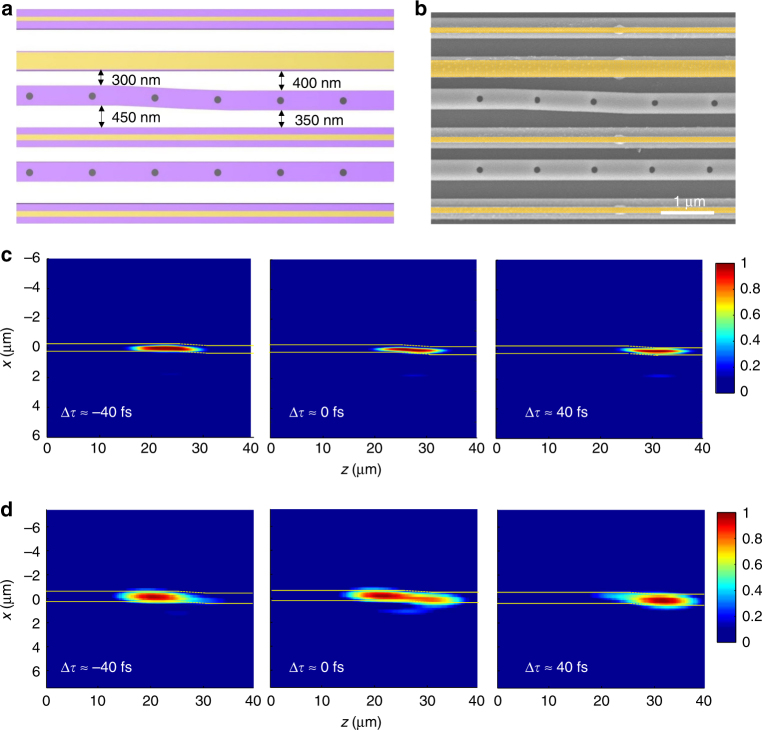
Experimental validation of the robustness of the zero mode. **a** The interface waveguide is adiabatically shifted 100 nm toward the PT symmetric semi-lattice over a distance of 5 µm in the *z* direction, reversing the local topological order around the interface from *t*_A_/*t*_B_ = 2.5 to $t_{\mathrm{A \prime}}/t_{\mathrm{B\prime}} = 0.8$. **b** SEM picture of the topology-transition region implemented in the Si waveguide array, where pseudo yellow color denotes the Cr depositions. The dimensions of each waveguide remain the same compared with the sample in Fig. 2c. **c** and **d** are numerically simulated (**c**) and experimentally (**d**) measured ultrafast dynamics of the zero mode to probe its robustness against topological disorders. Snapshots at different time delays show the pulse entering, propagating, and exiting around the topology-transition region

To test the robustness of the zero mode, the local topological perturbation is intentionally introduced to the interface waveguide, as shown in Fig. 4a. The interface waveguide is shifted by 100 nm toward the adjacent dimer in the PT symmetric semi-lattice, corresponding to the change of the local coupling strengths from $t_{\mathrm{A}}/t_{\mathrm{B}} = 2.5$ to $t_{\mathrm{A \prime}}/t_{\mathrm{B \prime}} = 0.8$. In this case, therefore, the local topological order is reversed. To enable the adiabatic transition between two opposite local topological orders, the shift of the interface waveguide gradually completes over a distance of 5 µm in the *z* direction. The zoom-in picture of the sample clearly confirms the implementation of this topology-transition region along the interface waveguide (Fig. 4b). Here we performed both numerical simulations (Fig. 4c) and heterodyne measurements (Fig. 4d) to characterize a wave packet propagation supported by the zero mode, showing the pulse entering, propagating, and exiting around the topology-transition region (Supplementary Movie 3). With the same single-waveguide excitation launched at the interface, the original zero mode is well formed in the interface waveguide. While the local topology varies after the wave packet enters the transition region, the zero mode persists with strong light localization at the interface. It is clear that the shape and dispersion of the wave packet after exiting the transition region remain almost unaffected, indicating the robust light transport carried by the zero mode. This is because the zero mode is protected under the PT symmetry invariants, even though the local topological order is completely reversed. The quantitative evaluation in experiments further confirms the robustness of the zero mode: the average group and phase indices during the topology transition are approximately *n*_g_ = 3.97 and *n*_eff_ = 2.37, which are almost identical to their counterparts of the zero mode without any disorder and perturbation.

## Discussion

In summary, we have demonstrated a novel robust photonic zero mode in a PT symmetric optical lattice, spatially localized at the interface separating broken and unbroken PT phases with the same topological order. Ultrafast heterodyne measurements confirmed the creation of a photonic zero-energy mode and its robustness against a local topological disorder at the interface. Our experiment provides the first evidence on a photonic platform of robust zero-energy modes under the protection of PT broken to symmetry phase transition. Different from the traditional defect states in topological photonics, the zero-energy bound state at the interface is protected by a non-Hermitian particle–hole symmetry^39^.

Through non-Hermitian engineering, the interface state can reemerge as a dominant state in the passive system. The restoration of topologically protected defect states, in spite of a uniform topological order in the entire structure, is enabled by the spatial quantum phase transition and enhanced by non-Hermitian loss engineering in the semi-lattices. A consistent definition of topological phases in non-Hermitian systems was introduced in a recent study^41^, using dynamical phases instead of states of matter. Therefore, a PT phase transition, like the one occurring at an exceptional point, can be regarded as a topological phase transition. If one accepts such a generalized definition of topological phases, the zero-mode interface state sustained by the quantum phase transition and demonstrated in our experiments can be regarded as arising from the connection of two complex crystals with different non-Hermitian topological phases. Our results suggest that non-Hermitian lattice engineering can do much more than simply stabilize a Hermitian topological mode^22–24^: it can create a new type of topological state which would disappear in the Hermitian limit. Our strategic quantum phase manipulation thus provides a genuine new route toward the creation and manipulation of topological protected states in non-Hermitian photonics and beyond.

## Methods

### Design and fabrication of the sample

To identify the zero-energy mode, we couple a quasi-TE polarized wave packet centered at the wavelength of 1550 nm into the interface waveguide and measure the light transport dynamics over the propagation distance *z*. To make the light transport directly visualized in the far field, we intentionally introduced periodic hole patterns on top of the waveguide lattice, satisfying a phase matching condition that coincides with the effective wavelength of the guided mode to efficiently couple guided light into the upward direction (Fig. 2b). In experiments, the samples were patterned on an SOI platform using overlay electron beam lithography, followed by the Cr deposition to introduce the onsite losses and reactive ion etching to form the PT symmetric waveguide lattice with hole patterns. The diameter of each hole was chosen to be 150 nm, which provided a good balance between the upward coupling efficiency and the insertion loss. The scanning electron microscope picture of the fabricated sample is shown in Fig. 2c.

### Ultrafast characterization

The ultrafast measurement system is a modified femtosecond-laser-powered Mach–Zehnder interferometer with a variable reference arm controlled by a delay line. The schematic of the optical setup is shown in Supplementary Fig. 3. The spatiotemporal reconstruction of the pulse is achieved using the Fourier transform method. In the spectrum, the first order of the interference pattern reveals the spatial envelop of the pulse. The pulse distribution is reconstructed through the process of the spectrum cyclic shift, low pass filtering, and inverse Fourier transform.

### Theoretical consideration of the topological perturbation

Assume the right shift of the interface A site (or waveguide) causes the local changes of the intra-dimer and inter-dimer coupling strengths as $t_{\mathrm{A \prime}} = t_{\mathrm{A}} - t_1$ and $t_{\mathrm{B \prime}} = t_{\mathrm{B}} + t_2$, respectively, as depicted in Fig. 3a. The modified Hamiltonian of the system reads

4 $$H = \left[ {\begin{array}{*{20}{c}} \ddots & {t_{\mathrm{B}}} & 0 & 0 & {\mathinner{\mkern2mu\raise1pt\hbox{.}\mkern2mu \raise4pt\hbox{.}\mkern2mu\raise7pt\hbox{.}\mkern1mu}} \\ {t_{\mathrm{B}}} & {i\gamma _1} & t_{\mathrm{A}}{\prime} & 0 & 0 \\ 0 & t_{\mathrm{A}}{\prime} & 0 & t_{\mathrm{B}}{\prime} & 0 \\ 0 & 0 & t_{\mathrm{B}}{\prime} & {i\gamma _2} & {t_{\mathrm{A}}} \\ {\mathinner{\mkern2mu\raise1pt\hbox{.}\mkern2mu \raise4pt\hbox{.}\mkern2mu\raise7pt\hbox{.}\mkern1mu}} & 0 & 0 & {t_{\mathrm{A}}} & \ddots \end{array}} \right],$$

where *γ*_1_ and *γ*_2_ denote the onsite loss coefficients of both left and right A waveguides next to the interface waveguide. Based on Eq. (4), the eigen-spectrum with the topological perturbation can be obtained (Fig. 3b), showing the dependence on the strength of the perturbation.

### Data availability

The data sets within the article and Supplementary Information in the current study are available from the authors upon request.

## Electronic supplementary material

Supplementary Information

Description of Additional Supplementary Files

Supplementary Movie 1

Supplementary Movie 2

Supplementary Movie 3
