# Supplementary material for: Photonic zero mode in a non-Hermitian photonic lattice
Source: Nat Commun. 2018 Apr 3;9:1308. doi: 10.1038/s41467-018-03822-8 (PMC5882938; doi:10.1038/s41467-018-03822-8)
Supplement: Supplementary file 1 — Supplementary Information [file 41467_2018_3822_MOESM1_ESM.pdf]

Supplementary Information

Photonic Zero Mode in a Non-Hermitian Photonic Lattice

Mingsen Pan,[1,2†] Han Zhao,[3†] Pei Miao,[1,2] Stefano Longhi[4] & Liang Feng[1*]

[1]*Department of Materials Science and Engineering, University of Pennsylvania, Philadelphia, PA 19104, USA*

[2]*Department of Electrical Engineering, The State University of New York at Buffalo, Buffalo, NY 14260, USA*

[3]*Department of Electrical and Systems Engineering, University of Pennsylvania, Philadelphia, PA 19104, USA*

[4]*Dipartimento di Fisica, Politecnico di Milano and Istituto di Fotonica e Nanotecnologie del Consiglio Nazionale delle Ricerche, Piazza L. da Vinci 32, Milano I-20133, Italy*

[†]These authors contributed equally to this work.

*Email: fenglia@seas.upenn.edu

Supplementary Note 1: Berry phase and topological invariant of a passive non-Hermitian system. From a topological perspective, the winding number and Chern number etc. are often analyzed to determine the topological invariants in Hermitian systems. Although several attempts have been made to generalize such parameters to a non-Hermitian system[1-4], the topological invariants are still not well defined. However, the topological nature can be equivalently probed by the global Berry phase[5,6], which, in our case, corresponds to the summation of complex Berry phase in both lower and upper bands. In this section, we show that the Berry phase of our passive non-Hermitian system is equivalent to that in a gain/loss balanced system[5], and the topological nature characterized by the global Berry phase is insensitive to the introduced non-Hermiticity.

The Hamiltonian matrix for the passive Su-Schrieffer-Heeger (SSH) model is

$$H(k)=\begin{bmatrix} -i\gamma_{\mathrm{A}} & \rho(k) \\ \rho^*(k) & -i\gamma_{\mathrm{B}} \end{bmatrix}, \tag{1}$$

where $\rho(k)=t_{\mathrm{A}}+t_{\mathrm{B}}\exp(-iak)=\rho_k\exp(i\phi_k)$, and the system has pure loss γ_{A} and γ_{B} on A and B site respectively. The eigenvalues are:

$$\lambda_{\pm}=-i\gamma_0\pm\sqrt{\rho_k^2+(i\gamma)^2}\ , \tag{2}$$

where $\gamma_0=(\gamma_{\mathrm{A}}+\gamma_{\mathrm{B}})/2$ is the background loss, and $\gamma=(\gamma_{\mathrm{A}}-\gamma_{\mathrm{B}})/2$ is the loss contrast. The corresponding right eigenvectors are:

$$|\lambda_+\rangle=\left[\rho_k\exp(i\phi_k)\quad i\gamma+\sqrt{\rho_k^2+(i\gamma)^2}\right]^T, \tag{3}$$

$$|\lambda_-\rangle=\left[\rho_k\exp(i\phi_k)\quad i\gamma-\sqrt{\rho_k^2+(i\gamma)^2}\right]^T, \tag{4}$$

which can be simplified as

$$|\lambda_+\rangle=[\exp(i\phi_k)\sin(\gamma_k/2)\quad \cos(\gamma_k/2)]^T, \tag{5}$$

$$|\lambda_-\rangle=[\exp(i\phi_k)\cos(\gamma_k/2)\quad -\sin(\gamma_k/2)]^T, \tag{6}$$

where $\gamma_k=\arctan(\rho_k/i\gamma)$. Similarly, the left eigenvectors can be derived as

$$\langle u_+|=[\exp(-i\phi_k)\sin(\gamma_k/2)\quad \cos(\gamma_k/2)], \tag{7}$$

$$\langle u_-|=[\exp(-i\phi_k)\cos(\gamma_k/2)\quad -\sin(\gamma_k/2)]. \tag{8}$$

We define the Berry connection matrix as

$$A = i\begin{bmatrix} \langle u_+ | d/dk | \lambda_+ \rangle & \langle u_+ | d/dk | \lambda_- \rangle \\ \langle u_- | d/dk | \lambda_+ \rangle & \langle u_- | d/dk | \lambda_- \rangle \end{bmatrix}, \quad (9)$$

in which the diagonal entries are the Berry connections for the top and bottom bands respectively. The Berry phase for each band can be calculated with the integration along one complete loop in the momentum space[7] $\varphi_{\mathrm{B}}^{\pm} = \oint_k i\langle u_\pm | \frac{d}{dk} | \lambda_\pm \rangle dk$, which, in our case, is,

$$\varphi_{\mathrm{B}}^{\pm} = \frac{\varphi_0}{2} \pm \frac{1}{2}\oint_{\varphi_k} \cos\gamma_k d\phi_k. \quad (10)$$

One can see Supplementary Equation (10) contains both the intrinsic Berry phase in the Hermitian limit (i.e. $\varphi_0/2$) and the non-Hermitian-induced geometric phase. Notice the background loss term γ_0 does not appear in the Berry phase calculations, and the system has identical Berry phases as the gain/loss balanced system[5].

The Berry phase of both bands are numerically calculated and are plotted in Supplementary Figure 1a, which clearly shows the evolutions of the Berry phase in each band with an increasing non-Hermiticity (i.e., loss contrast γ). In the Hermitian limit when $\gamma = 0$, the integrand $\cos\gamma_k$ vanishes, and the Berry phase plays the same role as the Zak phase[8], or winding number[9], whose value is quantized depending on the dimerization of the coupling ratio: either $\varphi_{\mathrm{B}}^{\pm} = 0$, if $t_{\mathrm{A}}/t_{\mathrm{B}} > 1$; or $\varphi_{\mathrm{B}}^{\pm} = \pi$, if $t_{\mathrm{A}}/t_{\mathrm{B}} < 1$. When the system is PT-symmetric, (i.e., in Phase I, $0 < \gamma < \rho_{k,\min} = |t_{\mathrm{A}} - t_{\mathrm{B}}|$), the eigenvalues are real, hence $\cos\gamma_k = i\gamma/\lambda$ is purely imaginary and $\varphi_{\mathrm{B}}^{\pm}$ are imaginary (we assume for the sake of definiteness $t_{\mathrm{A}}/t_{\mathrm{B}} > 1$). When $\rho_{k,\min} < \gamma < \rho_{k,\max} = |t_{\mathrm{A}} - t_{\mathrm{B}}|$, the system is in a mixed PT symmetry and breaking phase (Phase II), and the Berry phases generally become complex due to the complex eigenvalues. When the loss contrast is increased above PT-breaking threshold (i.e., $\rho_{k,\max} < \gamma$), the PT-breaking nature results in purely imaginary eigenvalues, and thus real Berry phases.

We can figure out that, at the Phase I to III transition interface, the Berry phase changes from imaginary to real values, and the system goes through regions with totally distinct quantum phases.

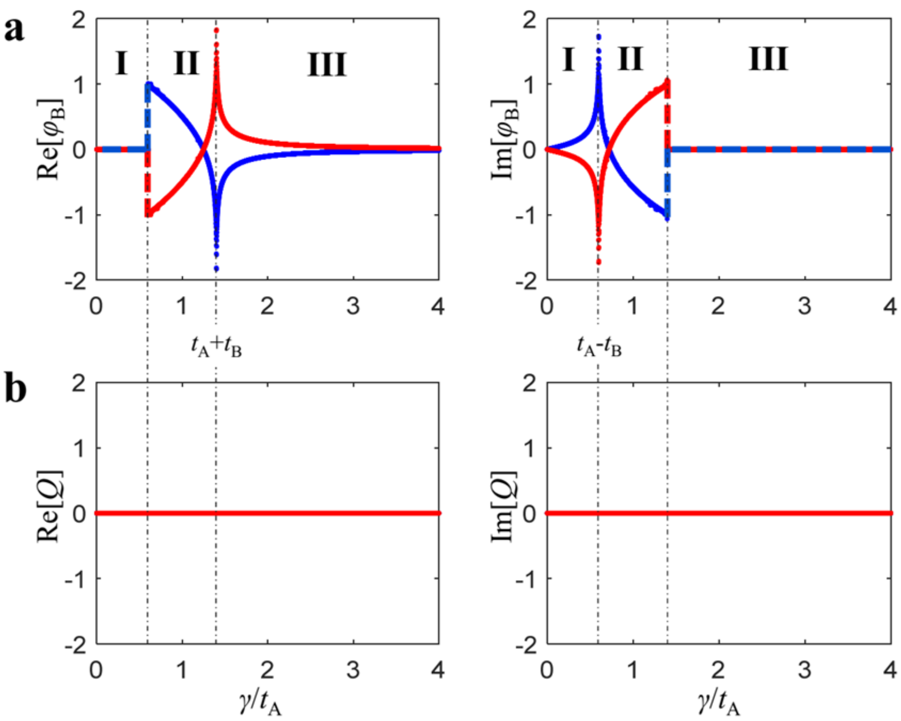

Supplementary Figure 1. Berry phase diagram of the system. **a** Real part and imaginary part of the Berry phase. The dashed-dotted vertical curves show the PT phase transition at the two critical points, $\gamma = t_A - t_B$, and $\gamma = t_A + t_B$. The red and blue curves are for the top and bottom bands respectively. **b** Real part and imaginary part of the global Berry phase for $t_A/t_B > 1$. Note that the global Berry phase is always vanishing in all the three phases regardless of non-Hermiticity.

To characterize the topological nature in this non-Hermitian system, we calculated the global Berry phase Q[10]:

$$Q = \oint_k \mathrm{tr}(A)dk = \varphi_0 \tag{11}$$

where $\mathrm{tr}(A)$ is the trace of Berry connection matrix A. The global Berry phase appears as a quantized value φ_0, depending on the dimerization of the SSH model, that is, the on-site non-Hermitian modulation has no impact on the global Berry phase. Hence, the topological nature of the SSH lattice is not changed by the induced non-Hermiticy. Here, we choose the dimerization of $t_A / t_B > 1$, which leads to the global Berry phase $Q = \varphi_0 = 0$ (Supplementary Figure 1b).

Supplementary Note 2: Eigenvalue analysis of the phase transition. Consider the interface formed by two SSH semi lattices mentioned in Note 1, shown in Supplementary

Figure 2a. For an infinite lattice, the corresponding eigenfunctions in the tight-binding limit are derived from the eigenvalue equations,

$$(\varepsilon - i\gamma_n^{\mathrm{A}})a_n = t_{\mathrm{B}}b_n + t_{\mathrm{A}}b_{n-1} \tag{12}$$

$$(\varepsilon - i\gamma_n^{\mathrm{B}})b_n = t_{\mathrm{B}}a_n + t_{\mathrm{A}}a_{n+1} \tag{13}$$

where n is the unit cell index, a_n and b_n are the mode amplitudes on waveguides A and B in the n-th unit cell, respectively, and γ_n^{A}, γ_n^{B} are the corresponding loss parameters. In each semi-array, the eigenvalues of the wave functions under uniform loss modulation γ_n^{A} and γ_n^{B} can be expressed as

$$\varepsilon_{\pm}(q) = -i(\gamma^{\mathrm{A}} + \gamma^{\mathrm{B}})/2 \pm \sqrt{t_{\mathrm{A}}^2 + t_{\mathrm{B}}^2 - 2t_{\mathrm{A}}t_{\mathrm{B}}\cos(q) - (\gamma^{\mathrm{A}} - \gamma^{\mathrm{B}})^2/4} \tag{14}$$

where $-\pi \le q < \pi$ is the Bloch wave number. With this modulation of loss contrast $\gamma^{\mathrm{A}} - \gamma^{\mathrm{B}}$, the sub-lattice can be shaped into the three different phases:

1) Phase I (unbroken passive PT phase): When the loss modulation satisfies the following condition

$$\left|\gamma^{\mathrm{A}} - \gamma^{\mathrm{B}}\right| < 2(t_{\mathrm{A}} - t_{\mathrm{B}}). \tag{15}$$

This phase corresponds to $\mathrm{Im}(\varepsilon_{\pm}(q)) = -i\left(\gamma^{\mathrm{A}} + \gamma^{\mathrm{B}}\right)/2$, independent of q in the entire Brillouin zone, and a gapped behavior of $\mathrm{Re}(\varepsilon_{\pm}(q))$ (right panel, Supplementary Figure 2b).

2) Phase II (partially broken passive *PT* phase): This phase occurs for

$$2(t_{\mathrm{A}} - t_{\mathrm{B}}) < \left|\gamma^{\mathrm{A}} - \gamma^{\mathrm{B}}\right| < 2(t_{\mathrm{A}} + t_{\mathrm{B}}), \tag{16}$$

which corresponds to a partially gapless behavior of $\mathrm{Re}(\varepsilon_{\pm}(q))$.

3) Phase III (fully broken passive PT phase): This phase occurs when the loss modulation is high enough to overcome the total inter-dimer and intra-dimer hopping amplitudes, i.e.,

$$\left|\gamma^{\mathrm{A}} - \gamma^{\mathrm{B}}\right| > 2(t_{\mathrm{A}} + t_{\mathrm{B}}), \tag{17}$$

with no real energy dispersion, $\mathrm{Re}(\varepsilon_{\pm}(q)) = 0$ (left panel, Supplementary Figure 2b).

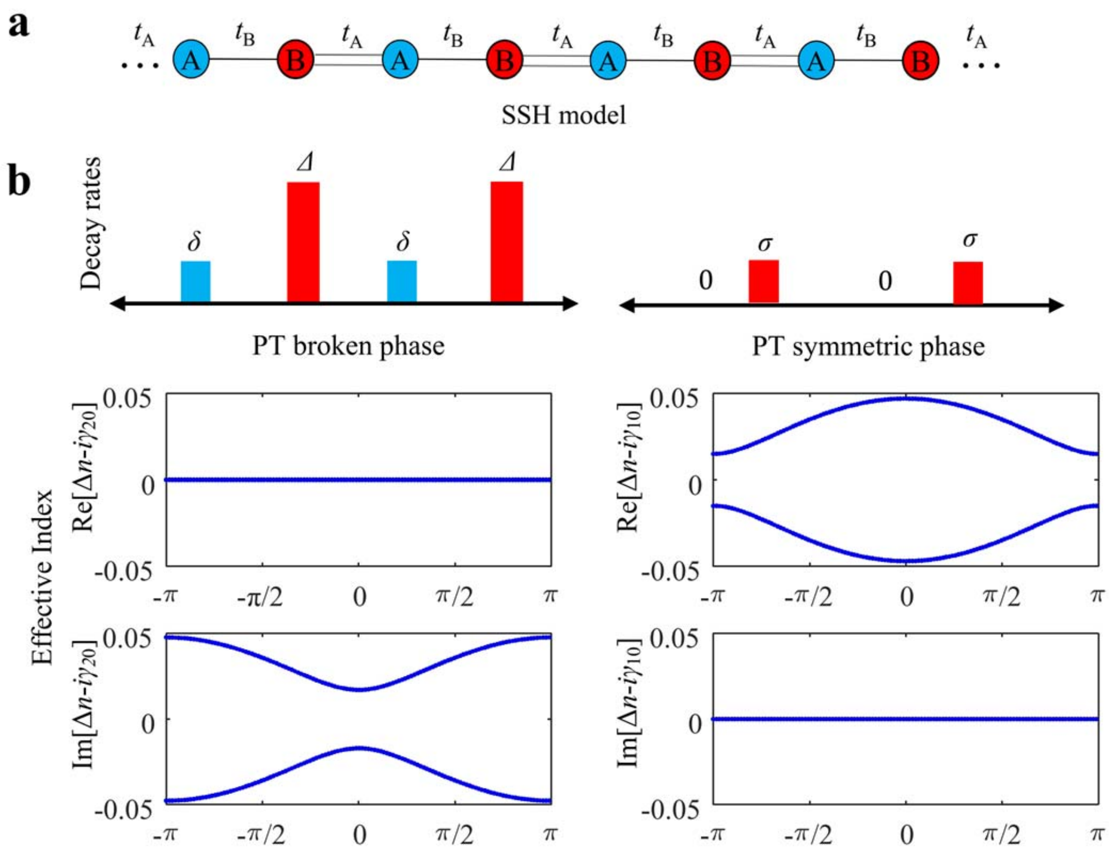

Supplementary Figure 2. Band structures of the non-Hermitian SSH model. a Schematic of a passive SSH model with a topological order indicated by $t_A > t_B$, in which the B sites in red denote higher loss and the A sites in light blue denote lower loss. **b** Loss contrast configurations and associated band structures normalized after filtering the average background loss terms: $\gamma_{20} = (\Delta + \delta)/2$ and $\gamma_{20} = \sigma/2$ for PT broken phase and PT symmetric phase, respectively. The left panels show the purely imaginary dispersion curve of the PT broken semi-lattice, while the right two panels show a symmetric energy splitting (lattice minibands) in the real spectrum for the right PT symmetric semi-lattice.

Zero-energy states induced by the PT phase transition.

Phases I and III in our system have totally different energy dispersions. Phase I has a real energy dispersion, while phase III has a purely imaginary energy dispersion. A spatial phase transition is realized by interfacing together two lattices in different phases. As shown in the Figures 1b and 1c in the main text, the topological nature of defect states can re-emerge at the phase I-III interface, enhanced by the non-Hermiticity-controlled phase transition. To realize the phase transition, we assume specifically the following loss configurations (Supplementary Figure 2b):

$$\gamma^{\mathrm{A}}=\begin{cases}\delta, n\le -1\\ 0, n\ge 0\end{cases},\ \gamma^{\mathrm{B}}=\begin{cases}\varDelta, n\le -1\\ \sigma, n\ge 0\end{cases} \tag{18}$$

with $\varDelta>\delta$. Any bound state, localized near the interface, is of the following form:

$$a_n=\begin{cases}A_{\mathrm{R}}(-1/X_1)^n, n\ge 0\\ A_{\mathrm{L}}(-X_2)^n, n\le -1\end{cases},\ b_n=\begin{cases}B_{\mathrm{R}}(-1/X_1)^n, n\ge 0\\ B_{\mathrm{L}}(-X_2)^n, n\le -1\end{cases}, \tag{19}$$

with $X_1=\exp(\mu_1)$, $X_2=\exp(\mu_2)$ and $\mathrm{Re}(\mu_{1,2})>0$ for localization.

Suppose $\varepsilon=-iE$ is the energy of an interface state satisfying Supplementary Equations (12) and (13). The connection of the two dimers at the interface (i.e. n = 0, 1) yields algebraic equations for X_1, X_2, and E:

$$E=\sigma/2\pm\sqrt{(\sigma/2)^2+t_{\mathrm{A}}t_{\mathrm{B}}(X_1+1/X_1)-t_{\mathrm{A}}^2-t_{\mathrm{B}}^2}, \tag{20}$$

$$E=(\varDelta+\delta)/2\pm\sqrt{((\varDelta-\sigma)/2)^2+t_{\mathrm{A}}t_{\mathrm{B}}(X_2+1/X_2)-t_{\mathrm{A}}^2-t_{\mathrm{B}}^2}, \tag{21}$$

$$-E(\delta-E)^2+t_{\mathrm{A}}(t_{\mathrm{B}}/X_1-t_{\mathrm{A}})(\delta-E)+Et_{\mathrm{B}}(t_{\mathrm{B}}-t_{\mathrm{A}}/X_2)=0. \tag{22}$$

The acceptable roots are those with $\left|X_{1,2}\right|>1$ for localization. A zero-energy mode corresponds to a real value of E, (i.e., $\mathrm{Re}(\varepsilon)=0$). This mode dominates over the extended (scattering) modes provided that the decay rate E is smaller than the decay rate of any extended (Bloch) mode in each semi-lattice. We note that, as compared to previous analysis[5], in our setup the average loss in the two semi-lattices is unbalanced, which substantially changes the existence domain and number of bound states. Extended numerical simulations show that, depending on the values of parameters, Supplementary Equations (20)-(22) can have zero roots (no interface modes); one root with energy $\mathrm{Re}(\varepsilon)=0$, corresponding to one zero-energy interface mode; or two roots with energies ε_1 and ε_2, corresponding to either two zero-energy modes $\mathrm{Re}(\varepsilon_1)=\mathrm{Re}(\varepsilon_2)=0$ with different intensity distributions and decay rates or two non-zero energy interface modes with eigen-energies $\varepsilon_2=-\varepsilon_1^*$, (i.e. $\mathrm{Re}(\varepsilon_1)=-\mathrm{Re}(\varepsilon_2)\neq 0$), and with the same intensity distribution and decay rate. For fixed values of the ratios $t_{\mathrm{A}}/t_{\mathrm{B}}>1$ and $\sigma/t_{\mathrm{B}}>0$, the following rather general results can be observed:

1) A necessary condition for an interface state to arise is that $\delta\neq 0$, i.e., some losses on sites A of the left semi-array are required.

2) Non-zero energy interface states appear in couple and are never dominant modes, i.e., their decay rate is larger than the one of extended (Bloch) modes.
3) For a given non-vanishing value of δ / t_{B}, there is a threshold value $\Delta_{\mathrm{th}} / t_{\mathrm{B}}$ for one dominant zero-energy mode to emerge, with $\Delta_{\mathrm{th}} / t_{\mathrm{B}} \to \infty$ as $\delta / t_{\mathrm{B}} \to 0$.

By choosing a group of non-Hermitian parameters to shape the bifurcation diagram, we can restore a robust zero-energy state to be dominant in the system, whose topological protective nature is enhanced by non-Hermiticity. This is discussed in the main text, Figures 1b and 1c.

Supplementary Note 3: Optical setup.

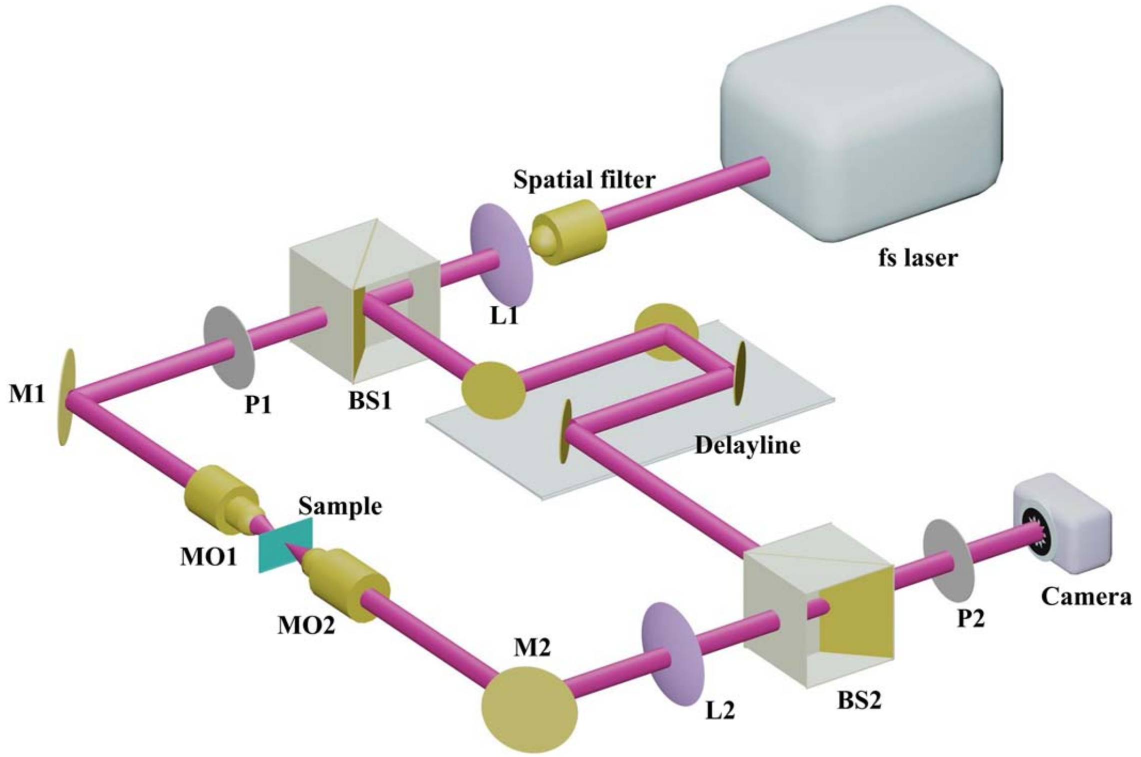

Supplementary Figure 3. Schematic of the optical setup for the ultrafast experiments. The modified Mach Zehnder interferometer is equipped with a delayline in the reference arm to measure the spatiotemporal information of the ultrafast pulse generated by a femtosecond (fs) laser. The sample is positioned in the signal arm to reconstruct the spatial information of the pulse in the waveguides. The group and phase velocities are measure within the temporal coherence of the fs laser by sweeping the delayline. Abbreviations for the optical components are: lens (L), beam splitter (BS), polarizer (P), mirrors (M), and micro-objects (MO).

As shown in Supplementary Figure 3, the interferometer is powered by an ultrafast laser source. The spatial filter and a 5-cm lens L1 produce a high-quality collimated beam split into the two arms by a 50:50 beam splitter (BS1). In the signal arm, a polarized light wave from polarizer P1 focuses on the input device on the sample by a micro objective (MO1 10X), which excites pulse propagation and radiates signal light waves from the circuits. The radiated spatial intensity image from the circuits is magnified by the imaging system (MO2 50X and 40-cm lens L2) with a resolution of 1 μm. The reference arm controls the optical path and creates a variable time delay for the pulse to reach the signal light wave. The light waves from the two arms mix together at BS2. To eliminate the influence of the incident light, a polarizer P2 that is orthogonal to P1 is installed before the camera. At the end of the setup, the total intensity is captured by the camera, which is a charge-coupled device (CCD), sensitive to the intensity distribution of the infrared light.

Supplementary Note 4: Wave propagation in a lossless array. The confinement of light in a tight-binding binary waveguide array is notoriously hard due to the evanescent coupling between the waveguides and discrete diffraction. To investigate the coupling characteristics of the waveguide array, we studied the lossless array of our proposed structure.

We define a wave packet with a temporal full width at half maximum (FWHM) of 162 fs and a center wavelength of 1550 nm to simulate the ultrafast laser source we used in the experiments. The wave packet is coupled into the middle waveguide in the array to excite a fundamental TE mode. The numerical simulation results at three delay times are shown in Supplementary Figure 4a. The middle waveguide locates at x = 0 μm, with the PT symmetric lattice below the middle waveguide and the PT symmetry-breaking lattice above the middle waveguide.

At zero delay time, the pulse coupled into the middle waveguide excites a wave packet propagation. In the experiments, the lossless array is tested with the heterodyne probing. The reconstructed pulse distributions at the three delay times are shown in Supplementary Figure 4b. The pulse intensity gradually spreads into the neighboring waveguides,

exciting bulk states in the lattice. At the end of the array, the coupling length of the pulse reaches to the third dimers in the lattices (1.55 μm for one dimer).

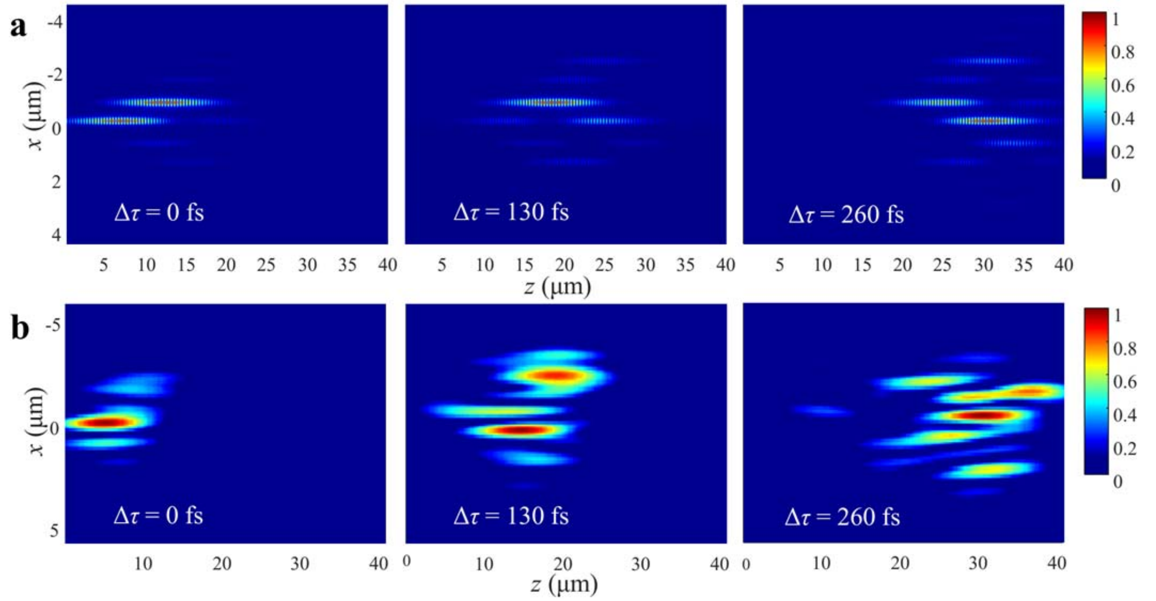

Supplementary Figure 4. Wave packet propagating in a lossless array. a Ultrafast simulation of wave packet amplitudes travelling in a lossless array at 0 fs, 130 fs, and 260 fs, respectively. **b** Experimentally reconstructed field amplitude distributions at the three time delays.

Supplementary References

1. Esaki, K., Sato, M., Hasebe, K., & Kohmoto, M. Edge states and topological phases in non-Hermitian systems. *Phys. Rev. B*, **84**, 205128. (2011).
2. Schomerus, H. Topologically protected midgap states in complex photonic lattices. *Opt. Lett.* **38**, 1912. (2013).
3. Poli, C., Bellec, M., Kuhl, U., Mortessagne, F., & Schomerus, H. Selective enhancement of topologically induced interface states in a dielectric resonator chain. *Nat. commun.*, **6**, 6710. (2015).
4. Weimann, S. et al. Topologically protected bound states in photonic parity-time-symmetric crystals. *Nat. Mater.* **16**, 433-438 (2017).
5. Zhao, H., Longhi, S. & Feng, L. Robust Light State by Quantum Phase Transition in Non-Hermitian Optical Materials. *Sci. Rep*. **5**, 17022 (2015).

6. Liang, S. D., & Huang, G. Y. Topological invariance and global Berry phase in non-Hermitian systems. *Phys. Rev. A* **87**, 012118 (2013).
7. Nenciu G. & Rasche, G. On the adiabatic theorem for non-self-adjoint Hamiltonians. *J. Phys. A* **25,** 5741–5751 (1992).
8. Zak, J. Berry's phase for energy bands in solids. *Phys. Rev. Lett*. **62**, 2747 (1989)
9. Zeuner, J. M., et. al. Observation of a topological transition in the bulk of a non-Hermitian system. *Phys. Rev. Lett.* **115**, 040402 (2015).
10. Liang, S. D., & Huang, G. Y. Topological invariance and global Berry phase in non-Hermitian systems. *Phys. Rev. A,* **87**, 012118 (2013).
