# Supplementary material for: Photonic zero mode in a non-Hermitian photonic lattice
Source: Nat Commun. 2018 Apr 3;9:1308. doi: 10.1038/s41467-018-03822-8 (PMC5882938; doi:10.1038/s41467-018-03822-8)
Supplement: Supplementary file 2 — Description of Additional Supplementary Files [file 41467_2018_3822_MOESM2_ESM.pdf]

Description of Additional Supplementary Files

File Name: Supplementary Movie 1
Description: Pulse propagation of the photonic zero mode in a non-Hermitian lattice.

File Name: Supplementary Movie 2
Description: Pulse propagation in a single waveguide.

File Name: Supplementary Movie 3
Description: Pulse propagation of the photonic zero mode in a non-Hermitian lattice with a local topological perturbation.
